# *Microbiome*, demystifying the role of microbial communities in the biosphere

**DOI:** 10.1186/2049-2618-1-1

**Published:** 2013-01-09

**Authors:** K Eric Wommack, Jacques Ravel

**Affiliations:** 1 Department of Plant and Soil Sciences, Delaware Biotechnology Institute, University of Delaware, Newark, DE, USA; 2 Institute for Genome Sciences and Department and of Microbiology and Immunology, University of Maryland School of Medicine, Baltimore, MD, USA

## 

The backstory to the motivation for launching *Microbiome* is a tale of two subdisciplines finding philosophical common ground fueled by substantial technological advancements in DNA sequencing and analysis. Until recently it was possible to neatly divide microbiology into two largely exclusive subdisciplines: clinical microbiology and environmental microbiology. Guided by Koch’s postulates for more than a century [1,2], clinical microbiologists have excelled in connecting specific microorganisms with disease in animals and plants, and through basic research have suppressed infectious diseases – saving countless lives through disease treatment and prevention and greater food security. Motivated initially by the desire to exploit the biochemical capabilities of microorganisms, environmental microbiologists have uncovered the large diversity of physiological capabilities that allow microorganisms to thrive wherever there exists sufficient free energy to sustain a proton gradient and feed essential biochemical pathways with reducing power. Emergent from these research activities was the discovery of extraordinary diversity of microbial life on Earth that has forever altered our perception of the tree of life. A look back at the five-kingdoms tree illustrated on the inside cover of a circa 1984 freshman biology textbook [3] is clear evidence of how far we have come in our appreciation of microbial diversity; new understanding made possible through basic environmental microbiology research.

The new appreciation of microbial diversity and the tree of life was driven by molecular phylogenetic analysis of the small subunit ribosomal RNA gene [4]. Save the discovery of the microscope and Van Leeuwenhoek’s use of it to discover microorganisms in the 17th century, few other discoveries have been so transformative to the discipline of microbiology. Indeed, rRNA-based techniques have enabled environmental microbiologists to catalogue and census microbial populations, capabilities at the foundation of most ecological investigations. A great deal of research effort within environmental microbiology has focused on differentiating microbial populations and then obtaining precise estimates of the density and census of individuals within each discrete population. Temporal changes in a population census inform us about the growth rate of a population, while geographic or spatial changes in population census inform us about connections between environmental features and the niche partitioning of species. Scientific exploitation of this universally conserved gene has transformed microbial ecology from a primarily descriptive natural history discipline to a fully-fledged quantitative ecology discipline. It is difficult to picture where microbial ecology would be today without small subunit 16S rRNA based methods.

While environmental microbiologists were shaping and pressing forward the new discipline of microbial ecology, clinical microbiologists were shaping the genomics revolution through shotgun DNA sequencing and computational assembly of whole bacterial genomes [5]. Access to whole genome sequence data provided the high-resolution details necessary to understanding the genetic basis of bacterial pathogenesis, its evolutionary foundations, and new avenues to therapeutic treatments for disease. Nevertheless, these advancements rested entirely on access to cultivated microorganisms. The limitations of cultivation for characterizing microbial diversity or the structure of microbial communities have been well known to environmental microbiologists for nearly half a century [6]. Indeed, the fact that <10% of microbes within a typical environmental sample are amenable to cultivation was a driving motivation behind the application of molecular genetic approaches in microbial ecology. Environmental microbiologists also understood that most ecosystem services provided through microbial activities – for example, the nitrogen cycle – require interacting populations of bacteria with each population responsible for one-component chemical transformation. Thus, dissecting the chemical underpinnings of ecosystems inevitably demands community-scale microbiological approaches.

It's difficult to credit a singular event to the recent philosophical push for clinical microbiologists to investigate how host-associated microbial communities may influence the health and disease states of plants and animals, and as a consequence adopt the community scale approaches developed by environmental microbiologists. Certainly, the $115 million National Institutes of Health investment in the Human Microbiome project [7,8] helped to attract the best scientific minds and cutting-edge technology to the problem; however, we believe that interest in microbiome research has much deeper roots fueled by a genuine scientific curiosity in dissecting the inner workings of the invisible microbial realm. Certainly, the interaction of microbiology subdisciplines that is occurring through microbiome research will benefit scientific advancement in both the clinical and environmental research fields.

Our hope is that *Microbiome* will facilitate the cross-fertilization of ideas, research methods and analyses, and theory between clinical and environmental microbiologists exploring the emergent impacts of microbial communities on the ecosystems they inhabit. A focus on microbial communities will therefore be the glue connecting the studies published in *Microbiome*. Topics covered in the journal will address all aspects of the study of microbial communities, including (but not limited to): marker gene surveys; ‘-omics’ surveys (including metagenomic, metatransciptomic, metaproteomic, and metabolomic), bioinformatic and other analytical tools, and community theory. To help facilitate data sharing within the microbiome research community, we have developed a new article type – Microbiome Announcement – designed for initial reporting of datasets in advance of the more expansive bioinformatics analyses required of in-depth studies. Although brief in terms of text and figures, Microbiome Announcements will emphasize detailed reporting of research methodologies and metadata through supplemental materials. We feel strongly that such detailed methods reporting will help advance the field and enhance the scientific value of meta-omics data.

Further aiding the advancement of microbiome research will be the open-access publication polices of *Microbiome*. Once accepted, all articles published in *Microbiome* will be freely available through the journal web portal and within the major research publication databases such as PubMed Central in due course. Also, because authors, not the publisher, will hold the copyright to their work, they will be free to distribute published articles. The scientific and societal benefits of open-access publication are clear — open access allows for the fastest and broadest possible dissemination of new findings, features critical in a journal focused on such a rapidly advancing research field.

To help realize our core goal of bringing together environmental and clinical microbiologists who share interests in studying microbial communities, we have assembled an editorial board of scientists who span a broad range of research interests within environmental science, computational biology, medicine, and agriculture. We hope that you will join us in this exciting and novel endeavor to promote scientific cross-fertilization between clinical and environmental microbiology and advance fundamental understanding of microbial communities in all corners of the biosphere.
